# Hortensins, Type 1 Ribosome-Inactivating Proteins from Seeds of Red Mountain Spinach: Isolation, Characterization, and Their Effect on Glioblastoma Cells

**DOI:** 10.3390/toxins16030135

**Published:** 2024-03-04

**Authors:** Sara Ragucci, Veronica Russo, Angela Clemente, Maria Giuseppina Campanile, Maria Antonietta Oliva, Nicola Landi, Paolo Vincenzo Pedone, Antonietta Arcella, Antimo Di Maro

**Affiliations:** 1 Department of Environmental, Biological and Pharmaceutical Sciences and Technologies (DiSTABiF), University of Campania ‘Luigi Vanvitelli’, Via Vivaldi 43, 81100 Caserta, Italy; sara.ragucci@unicampania.it (S.R.); angela.clemente@unicampania.it (A.C.); mariagiuseppina.campanile@unicampania.it (M.G.C.); nicola.landi@unicampania.it (N.L.); paolovincenzo.pedone@unicampania.it (P.V.P.); 2 IRCCS Istituto Neurologico Mediterraneo ‘NEUROMED’, Via Atinense 18, 86077 Pozzilli, Italy; veronica.2306@hotmail.it (V.R.); olv78@hotmail.com (M.A.O.); 3 Institute of Crystallography, National Research Council of Italy, Via Vivaldi 43, 81100 Caserta, Italy

**Keywords:** *Atriplex hortensis* L., cytotoxicity, edible plants, red orache, rRNA N-glycosylases, U87MG glioblastoma cells

## Abstract

Ribosome inactivating proteins (RIPs) are specific N-β-glycosylases that are well-characterized in plants. Their enzymatic action is to damage ribosomes, thereby blocking protein translation. Recently, several research groups have been working on the screening for these toxins in edible plants to facilitate the use of RIPs as biotechnological tools and biopesticides and to overcome public prejudice. Here, four novel monomeric (type 1) RIPs have been isolated from the seeds of *Atriplex hortensis* L. var. *rubra*, which is commonly known as edible red mountain spinach. These enzymes, named hortensins 1, 2, 4, and 5, are able to release the β-fragment and, like many other RIPs, adenines from salmon sperm DNA, thus, acting as polynucleotide:adenosine glycosidases. Structurally, hortensins have a different molecular weight and are purified with different yields (hortensin 1, ~29.5 kDa, 0.28 mg per 100 g; hortensin 2, ~29 kDa, 0.29 mg per 100 g; hortensin 4, ~28.5 kDa, 0.71 mg per 100 g; and hortensin 5, ~30 kDa, 0.65 mg per 100 g); only hortensins 2 and 4 are glycosylated. Furthermore, the major isoforms (hortensins 4 and 5) are cytotoxic toward human continuous glioblastoma U87MG cell line. In addition, the morphological change in U87MG cells in the presence of these toxins is indicative of cell death triggered by the apoptotic pathway, as revealed by nuclear DNA fragmentation (TUNEL assay).

## 1. Introduction

Ribosome-inactivating proteins (RIPs) are toxic enzymes (N-β-glycosylases, EC 3.2.2.22), mainly isolated from flowering plants (angiosperms), which irreversibly damage ribosomes and cause cell death via the apoptotic pathway [1]. Their enzymatic activity consists in the ability to remove a single adenine from 28S rRNA of the large 60S ribosomal subunit (A_4324_ in rat liver ribosomes), which promotes structural changes in the ribosomes, leading to the inhibition of protein synthesis [2]. In particular, the specific apurinic site produced by the enzymatic activity of RIPs resides in the sarcin ricin loop (SRL), which changes its conformation, losing its ability to interact with eukaryotic or prokaryotic elongation factors (EF-2 or EF-G, respectively), thereby arresting mRNA–tRNA translocation [3]. In addition, RIPs exhibit the ability to remove adenines from poli(A), RNA, and DNA substrates, for which are known as polynucleotide:adenosine glycosylases (PNAG) [4,5].

Most RIPs can be distinguished into two main groups according to the absence or presence of a quaternary structure. Indeed, these toxins can be grouped into type 2 (~60 kDa and neutral pI) and type 1 (~30 kDa and basic pI) RIPs. Type 2 RIPs consist of a toxic chain (A-chain; N-β-glycosylase) linked to a cell-binding B chain (B-chain) through an interchain disulfide bond and hydrophobic interactions. The lectin activity of the B-chain is targeted toward galactose moieties on the mammalian cell surface and promotes the entrance of the A-chain into cells. Type 1 RIPs are characterized by the absence of the lectin B-chain, which significantly limits the access into the cells, thereby determining their lower cytotoxicity [6]. In addition, some noncanonical type 3 RIPs have been isolated from Poaceae with a C-terminal domain of unknown functionality (e.g., barley JIP-60) [7] or require proteolytic processing for enzymatic activation (e.g., maize b-32) [8].

In recent years, experimental evidence suggests a physiological role for plant RIPs in both stress processes [9,10] and defense against pathogens and insect pest attacks [11], although an exhaustive picture of their physiological action is not yet clear. On the other hand, the investigation on RIPs is of interest considering their possible application in biomedical applications and agriculture. Indeed, in medicine, several RIPs are linked to antibodies or other nanocarriers to obtain immunotoxins or nanoconjugates selectively toxic for harmful cells to be eliminated [12,13], while in agriculture, the modulation of their expression could improve the plant defense mechanisms toward viruses [14], fungi, and insects [15].

RIPs have been identified in more than 17 families of angiosperms, non-retrieved in gymnosperms, and most of these enzymes are purified from Cucurbitaceae (Cucurbitales order), Euphorbiaceae (Malpighiales order), Phytolaccaceae (Caryophyllales order), Poaceae (Poales order), Rosaceae (Rosales order), and Adoxaceae (Dipsacales order) families [16], while in silico analysis confirms the presence of RIP genes in Eudicots, Monocots, and Magnoliids [17,18].

Because many RIPs have been isolated from the Amaranthaceae family (Caryophyllales order), our research group has recently characterized several type 1 RIPs from Amaranthaceae (i.e., quinoin from the seeds of *Chenopodium quinoa* L. [19] and sodins from the seeds, roots, and leaves of *Salsola soda* L. [20]), while other groups have isolated type 1 RIPs from this family [21] (e.g., beetins from *Beta vulgaris* L. [22], amaranthin from *Amaranthus viridis* L. [23], and SoRIP1 from *Spinacia oleracea* L. [24]). The characterization of RIPs in Amaranthaceae could have significant applicative implications considering that most of the species from this family are consumed like vegetables (e.g., spinach), while quinoa seeds are considered pseudocereals, being a rich source of crude proteins and nutraceutical compounds [25].

*Atriplex hortensis* L., also known as orache or mountain spinach, is a species belonging to the Amaranthaceae family, which is known for drought and salt tolerance and can grow in otherwise inhospitable soils [26]. The seeds and leaves of *A. hortensis* are edible and have been consumed since ancient times (good source of crude quality protein and vitamins) [27]. In addition, the extracts of *A. hortensis* are used in traditional medicine as a health tonic and diuretic; moreover, they are efficacious in the treatment of gout and tumors [28,29].

In this framework, the aim of the present investigation is to increase the knowledge on the distribution of RIPs in the Amaranthaceae family, verifying the presence of these enzymes in *A. hortensis* var. *rubra* (red mountain spinach). Therefore, we report for the first time the purification of four novel type 1 RIPs (named hortensins) from red mountain spinach seeds. In addition, the enzymatic and physicochemical characterization, as well as the cytotoxic effect of the major isoforms (hortensins 4 and 5) against U87MG glioblastoma cell line were performed.

## 2. Results and Discussion

### 2.1. Isolation of Hortensins

Crude extracts from *A. hortensis* seeds inhibited protein synthesis by a rabbit reticulocyte lysate system (IC_50_ = 516.0 ng protein/mL) and exhibited depurination activity on salmon sperm DNA (3.0 µg of protein crude extract led to an increase in absorbance from ~0.025 to ~0.133 compared to same amount BSA at 260 nm). To verify whether the activity was due to the presence of type 1 RIPs, a purification procedure reported in the methods was performed. Total soluble proteins were extracted from *A. hortensis* seeds in phosphate-buffered saline and acid precipitated with acetic acid (pH 4.0).

Soluble proteins were fractionated using the following steps: (i) cation exchange chromatography on SP-Streamline; (ii) gel filtration chromatography; and (iii) cation exchange chromatography on CM-Sepharose.

**Figure 1:**
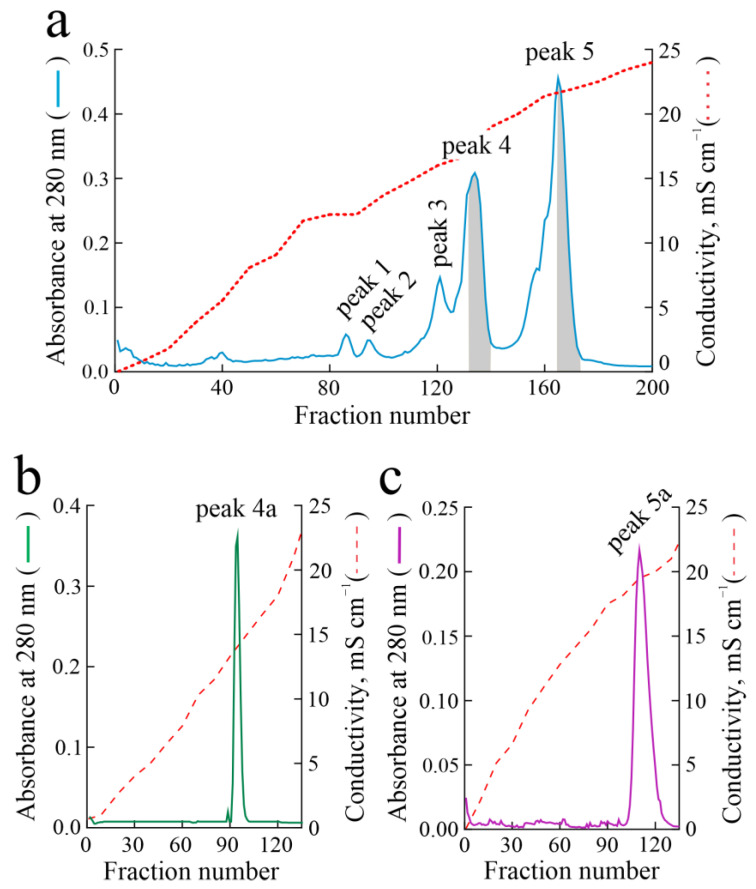
Purification of type 1 RIPs from the seeds of *Atriplex hortensis* var. *rubra*. (**a**) Elution profile from the CM-Sepharose chromatography (volume fraction: 5.0 mL); (**b**) S-Sepharose re-chromatography of peak 4 pooled fractions from chromatographic profile in (**a**) highlighted in grey; (**c**) S-Sepharose re-chromatography of peak 5 pooled fractions from chromatographic profile in (**a**) highlighted in gray. The volume fraction of S-Sepharose re-chromatography was 4.5 mL.

Five protein peaks (numbered from 1 to 5) were obtained from the last purification step (cation exchange chromatography on CM-Sepharose; Figure 1a), all able to inhibit protein synthesis by a rabbit reticulocyte lysate system.

SDS-PAGE analysis (Figure S1) showed that peak 1 of Figure 1a contained a single protein band with a molecular weight of ~29.5 kDa (lanes 84, 86 and 88), while peak 2 contained a major protein band of ~29 kDa (lanes 92, 94 and 97) representing ~95% of the total proteins (Figure S2). Peaks 3 and 4 showed the presence of at least two major protein bands with molecular weights of ~28.5 and ~20.4 kDa (lanes of peak 3: 118, 121, and 123; lanes of peak 4: 129 and 131–138). Finally, peak 5 showed a major protein band with a molecular weight of ~30 kDa and two minor protein bands of ~19.4 and ~18.0 kDa (lanes 152, 154, and 156–171).

Therefore, the protein fractions of peaks 1 and 2 were pooled, dialyzed against deionized water, concentrated, and named hortensins 1 (29.5 kDa; yield ~0.28 mg/100 g of seeds) and 2 (29 kDa; yield ~0.29 mg/100 g of seeds), respectively. On the other hand, to improve the purity of major proteins contained in peaks 4 and 5, the fractions corresponding to the decreasing part (right side highlighted in gray) of both peaks 4 and 5 (Figure 1a) were further fractionated using cation exchange chromatography on S-Sepharose, considering their heterogeneity. Fractions 94–96 of the single peak (peak 4a; Figure 1b) obtained after peak 4 right-side fraction re-chromatography contained a single protein band (~28.5 kDa) with a yield of ~0.71 mg/100 g of seeds, hereafter named hortensin 4. Fractions 115–130 of the single peak (peak 5a; Figure 1c) obtained after peak 5 right-side fraction re-chromatography contained a single protein band (~30 kDa) with a yield of ~0.65 mg/100 g of seeds, hereafter named hortensin 5. In light of this, the protein fractions containing hortensis 4 and 5 were pooled, dialyzed against deionized water, and concentrated. On the contrary, protein fractions obtained using S-Sepharose re-chromatography of peak 3 from CM-Sepharose did not allow us to obtain pure proteins although peak fractions showed depurination activity on salmon sperm DNA.

**Figure 2:**
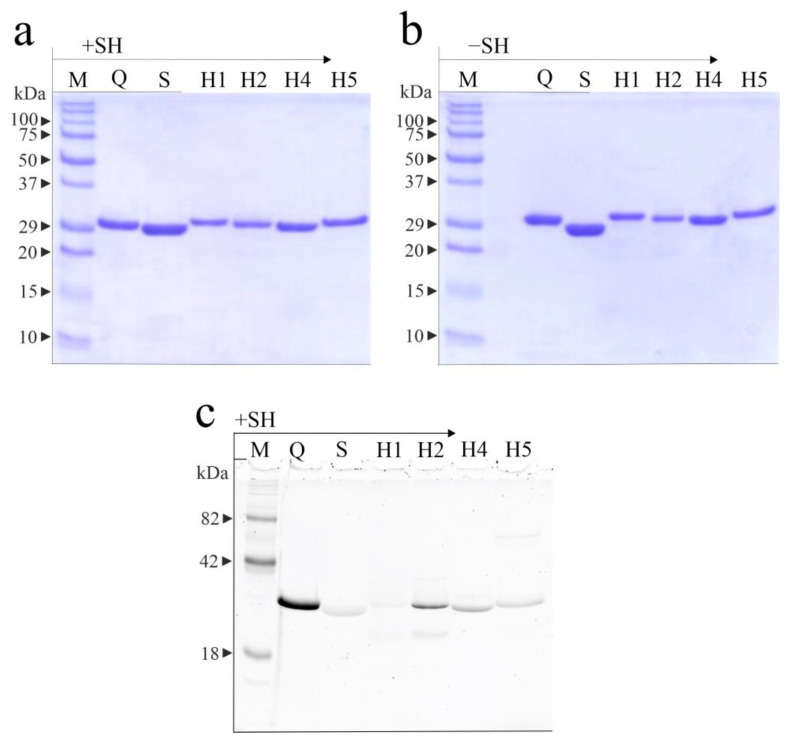
SDS-PAGE analysis of hortensins 1, 2, 4, and 5 (3.0 µg) from seeds of *Atriplex hortensis* var. *rubra* (lanes H1, H2, H4, and H5, respectively) with (**a**; +SH) and without (**b**; −SH) β-mercaptoethanol. M, molecular weight markers, lanes Q and S, quinoin (type-1 RIP from the seeds of *Chenopodium quinoa*; 3.0 µg), and sodin 5 (type-1 RIP from the seeds of *Salsola soda*; 3.0 µg), respectively. SDS-PAGE was performed on 12% polyacrylamide gel visualized with Coomassie brilliant blue staining. In (**c**), in-gel glycan detection of hortensins 1, 2, 4, and 5 (3.0 µg) using the Pro-Q Emerald 300 glycoprotein staining kit after SDS-PAGE separation. Stained glycoproteins were visualized using UV transillumination. M, glycosylated molecular weight markers, lane Q, quinoin (N-glycosylated type-1 RIP; 3.0 µg), and lane S, sodin 5 (non-glycosylated type 1 RIP; 3.0 µg).

The purity and integrity of hortensins 1, 2, 4, and 5 were confirmed using SDS-PAGE in the presence or absence of β-mercaptoethanol (Figure 2a,b). In addition, two previously characterized type 1 RIPs were used as references: glycosylated quinoin isolated from the seeds of *C. quinoa* [19] and non-glycosylated sodin 5, isolated from the seeds of *S. soda* [20].

Finally, considering that several RIPs from plant seeds are glycosylated [30], a specific analysis for glycoprotein detection was conducted. When analyzed using SDS-PAGE and sugar staining under reducing conditions, the four hortensins appear differently glycosylated (Figure 2c). Indeed, it is evident that hortensin 1 is not glycosylated, hortensin 2 is highly glycosylated, hortensin 4 is faintly glycosylated, while hortensin 5 appears not glycosylated, like sodin 5.

### 2.2. Enzymatic Features of Hortensins 1, 2, 4, and 5

Purified hortensin 1, 2, 4, and 5 inhibited protein synthesis in a rabbit reticulocyte lysate system, with IC_50_ values of 31.83 ± 1.30 pM (~0.92 ng/mL), 25.33 ± 1.32 pM (~0.73 ng/mL), 11.47 ± 0.78 pM (~0.33 ng/mL), and 29.18 pM (~0.85 ng/mL), respectively. These values are similar to the IC_50_ of sodins isolated from *S. soda* tissues (IC_50_ = 4.83–79.31 pM) [20] and fall within the range of most of known type 1 RIPs (IC_50_ = 10–4000 pM) [6].

In light of this, to classify hortensins 1, 2, 4, and 5 as specific N-β-glycosylases (EC: 3.2.2.22) and, thus, as novel members of the type 1 RIPs family, considering the absence of a quaternary structure (molecular weight of ~30 kDa), we decided to verify whether hortensins are able to release the β-fragment (hallmark of RIPs) when incubated with rabbit ribosomes [2]. Considering this, Endo’s assay was conducted. This assay allows to detect the hydrolysis of the N-glycosidic bond between a specific adenine (A_4324_ in rat liver 28S rRNA) and its ribose in ribosomes. This activity can be highlighted using polyacrylamide gel electrophoresis, which detects the release of a specific RNA fragment (Endo’s fragment or β-fragment) following the incubation of the apurinic RNA in the presence of acid aniline [2].

**Figure 3:**
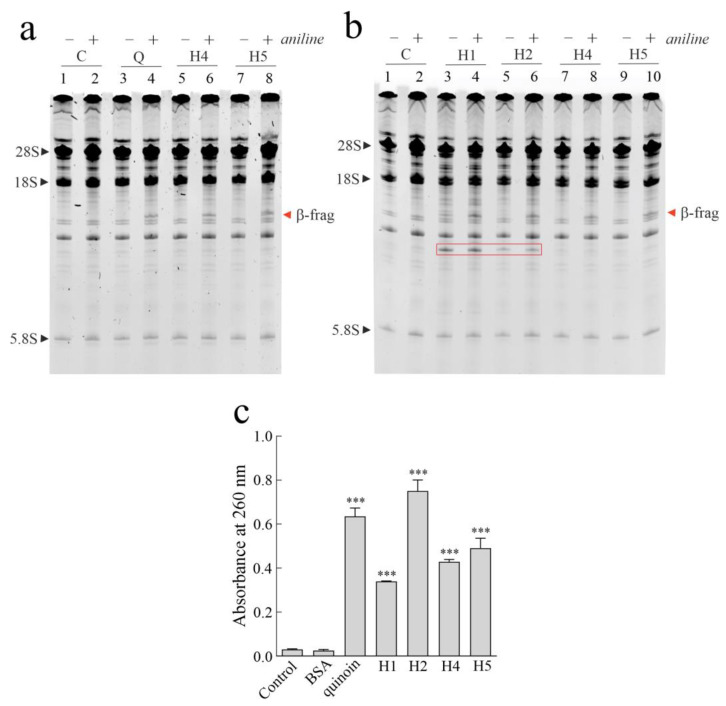
(**a**,**b**), rRNA N-β-glycosylase activity on rabbit ribosomes; 3.0 µg quinoin (Q; reference type 1 RIP from seeds of *C. quinoa*) hortensins 1 (H1), 2 (H2), 4 (H4), and 5 (H5) were incubated with ribosomes. The rRNA was extracted, subjected to aniline treatment, and separated, as reported in the Materials and Methods section; (+) and (−) indicate with and without aniline treatment, respectively. “β-frag” indicates the Endo’s fragment released following aniline treatment of rRNA from rabbit ribosomes. The red rectangle highlights the additional RNA fragment released after ribosomes incubation with hortensins 1 and 2 and detected with and without aniline treatment. (**c**) Polynucleotide:adenosine glycosylase activity of BSA (negative control), type 1 RIPs quinoin (positive control) and hortensins 1 (H1), 2 (H2), 4 (H4), and 5 (H5). Proteins (3.0 µg) were incubated with salmon sperm DNA, as reported in the Materials and Methods section. The mean ± SD results from three experiments performed in triplicate is shown. One-way ANOVA + post hoc Dunnett’s test (***, *p* < 0.001) was used to analyze data, with respect to the control.

As displayed in Figure 3a, without aniline treatment, no fragment was released when rabbit reticulocyte lysate was incubated with quinoin (used as reference type 1 RIP) from seeds of *C. quinoa* and hortensins 4 and 5 (major isoforms). However, in the presence of aniline, the specific β-fragment was released. Therefore, it is evident that hortensins 4 and 5 are N-glycosylases acting on the SRL of rabbit ribosomes. Subsequently, the same assay was repeated on the minor isoforms (hortensins 1 and 2), using the major isoforms as positive control (Figure 3b). It is evident that all hortensins are novel type 1 RIPs that release the β-fragment, which is a hallmark of RIPs. However, following the enzymatic action of hortensins 1 and 2 on rabbit ribosomes, the presence of an additional RNA fragment (see the red rectangle in Figure 3b) was evident with and without aniline treatment and was not detected after ribosome incubation with hortensins 4 and 5. To exclude the possible slight ribonuclease contamination, a ribonuclease zymography was performed. The zymogram reported in Figure S3 displays the absence of unspecific ribonuclease activity. However, a possible additional enzymatic action (e.g., nuclease) could justify the release of the additional fragment [6,31].

Furthermore, considering the polynucleotide:adenosine glycosylase activity (PNGA) detected during the purification of hortensins, a comparative assay was performed using 3.0 µg toxins (Figure 3c). Hortensin 2 displays the highest PNAG activity with respect to the other three hortensins (~1.5-, 1.9-, and 2.3-fold more active than hortensins 5, 4, and 1, respectively) and quinoin (~1.2-fold), the latter used as a reference enzyme [19].

### 2.3. Inhibiting Effect of Hortensins 4 and 5 on Cell Growth and Morphology of Glioblastoma U87MG Cells

**Figure 4:**
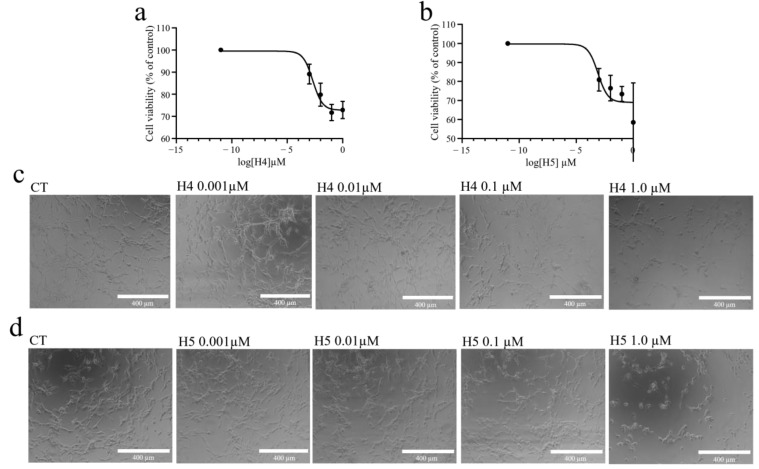
In (**a**,**b**), graphs showing the cell viability percentage (MTT assay) of U87MG cells as a function of varying concentrations (0.001, 0.01, 0.1, and 1.0 µM) of hortensin 4 (H4) and hortensin (H5), respectively, after 72 h of treatment. The control was assumed to be part of the dose–response curve, considering the very low concentration of 10^−11^ µM. Data were processed using GraphPad Prism and are reported as mean ± SD. In (**c**,**d**), representative images of U87MG cells with different concentrations (0.001, 0.01, 0.1, and 1.0 µM) of hortensin 4 (H4) and hortensin 5 (H5), respectively, after 72 h of treatment. Magnification 10×. Scale bar, 400 µm.

Considering the specific enzymatic action of hortensins 4 and 5 and the higher yield of purification with respect to hortensins 1 and 2, we decided to evaluate only the cytotoxic effects of the major isoforms (hortensins 4 and 5) on U87MG glioblastoma continuous cell line. Indeed, a previously published work reported that quinoin, isolated from *C. quinoa* seeds, exhibits high cytotoxicity towards U87MG and two primary cell lines established from patients’ gliomas (NULU and ZAR) [32]. As shown in Figure 4a,b, both hortensin 4 and 5 induced a decrease in cell viability in a concentration- and time-dependent manner, and the dose–response curves reached a plateau at high toxin tested doses. In addition, when administered to U87MG glioblastoma cells in culture, both hortensins 4 and 5 induced a significant change in the cell morphology. In particular, within the first 24 h after treatment, the cells treated with hortensins 4 and 5 at different concentrations (0.001, 0.01, 0.1, 1.0 µM) show rarefaction that is higher after 72 h of treatment (Figure 4c,d). Indeed, many spaces without cell colonies are visible in the plate and the morphology has completely changed; the cells are less distended and have small pyknotic nuclei.

### 2.4. Cell Growth and Viability of Glioblastoma U87MG Cells after Treatment with Hortensins 4 and 5

**Figure 5:**
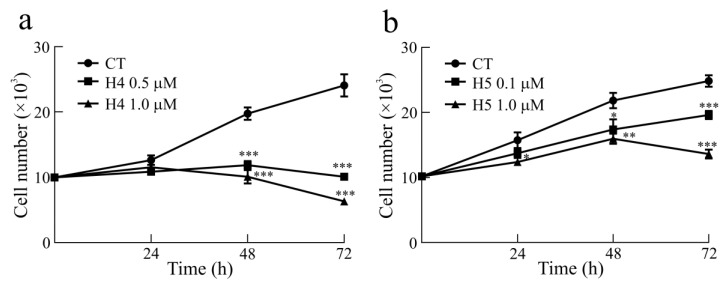
In (**a**,**b**), dose- and time-dependent growth inhibition curves of U87MG cells treated with 0.5 and 1.0 µM hortensin 4 (H4) and 0.1 and 1.0 µM hortensin 5 (H5) for 24, 48, and 72 h. All the values are the means ± SEM of individual determinations. Unpaired *t*-test, *p*-value < 0.05. According to statistical analysis, * *p*-value 0.01 to 0.05 (significant), ** *p*-value 0.001 to 0.01 (very significant), *** *p*-value 0.0001 to 0.001 (extremely significant).

According to the dose–response curves, we evaluated the effect of hortensins 4 and 5 on human glioma cells’ U87MG growth rate, using 0.5 and 1.0 µM of hortensin 4 or 0.1 and 1.0 µM of hortensin 5 daily for 3 days, starting 1 day after plating. Following the treatment, the linear phase of growth in the continuous cell line U87MG was reduced with the cell number already being substantially reduced at 1 day after treatment and increased after 2 and 3 days of treatment (Figure 5a,b).

### 2.5. TUNEL Assay of Glioblastoma U87MG Cells after Treatment with Hortensins 4 and 5

**Figure 6:**
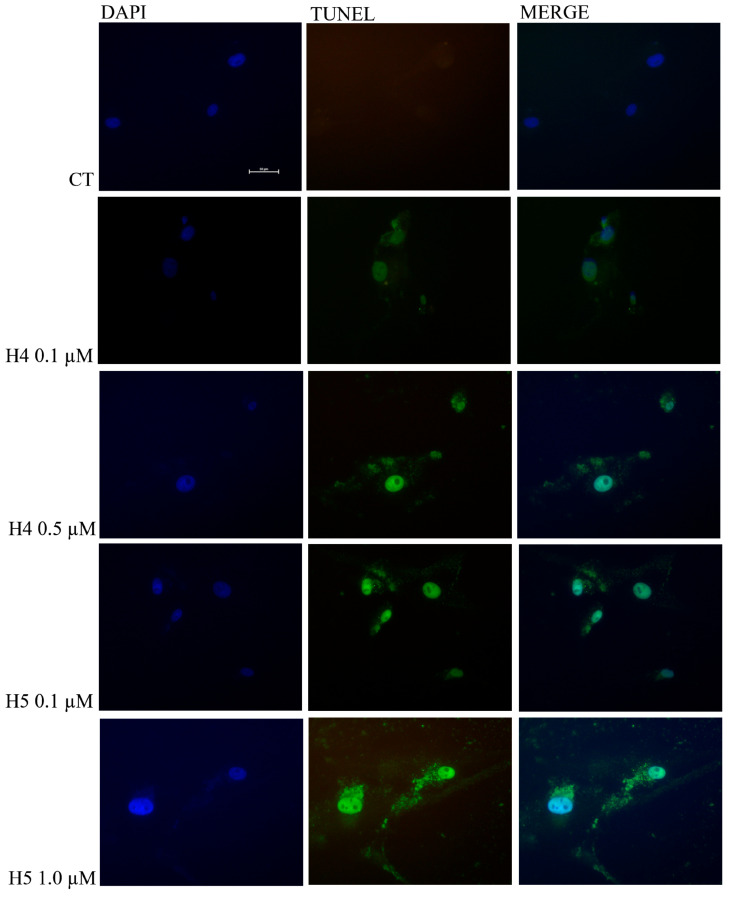
Representative images from three independent experiments (n = 3) after TUNEL assay of U87MG treated with hortensin 4 (H4; 0.1 and 0.5 μM) and hortensin 5 (H5; 0.1 and 1.0 μM) for 72 h compared with untreated U87MG (CT). Magnification 40×. Scale bar, 50 µm.

The morphological change in U87MG cells after treatment with hortensins 4 and 5, which was characterized by a rounded shape and shriveled nucleus, raised the hypothesis that the mechanism of growth inhibition might be the activation of apoptosis. This evidence is in agreement with previous studies, considering that most of RIPs cause cell death through apoptosis [1,6,13]. Therefore, we produced experimental evidence to demonstrate the involvement of this pathway in cell death mediated by hortensins by detecting the DNA breakage by labelling the free 3′-hydroxyl termini using terminal deoxynucleotidyl transferase-mediated dUTP nick end labelling (TUNEL). This assay is still commonly used as a marker of apoptotic cell death because genomic DNA breaks occur at both early and late phases of apoptosis. The purpose of this assay is to determine the cytotoxic and apoptotic effects of hortensins 4 and 5. To this aim, we performed a tunnel assay on U87MG cells treated with 0.1 and 0.5 µM hortensin 4 or 0.1 and 1.0 µM hortensin 5 for 72 h, Figure 6. The first row shows untreated U87MG cells used as a control. The second and third rows show cells treated with 0.1 and 0.5 µM hortensin 4, respectively, while the last two rows are cells treated with 0.1 and 1.0 µM hortensin 5 for 72 h. The increase in green fluorescent cells (second column, Figure 6), with respect to the control, indicates the presence of nuclear DNA fragmentation consistent with the induction of the apoptotic mechanism.

Finally, apoptotic cell counting was conducted in the larger total population; for each treatment, we counted positive cells in three different fields at 20× magnification (Figure S4). The percentage of apoptotic cells is about 8.0% and 11% in cells treated with 0.1 and 0.5 µM hortensin 4, respectively, and 11% and 50% in cells treated with 0.1 and 1.0 µM hortensin 5, respectively. On the other hand, in control cells (cells not treated with RIPs), the count of apoptotic cells was not performed because there was no evidence of apoptosis.

## 3. Conclusions

The seeds of edible red mountain spinach contain four type 1 RIPs, which we have named hortensins 1, 2, 4, and 5. These toxins have specific N-β-glycosylase activity on rabbit ribosomes and release adenines from salmon DNA (PNAG activity). In addition, the major isoforms (hortensins 4 and 5) are cytotoxic against U87MG glioblastoma cells, leading to cell death via the apoptotic pathway. The toxic effect on malignant cells highlights the potential use of hortensins in biomedicine by targeting their cytotoxicity as part of immunotoxins or nanoconstructs. Indeed, one of the biggest hurdles for researchers has been the development of a successful therapy for the treatment of glioblastoma, which is made more difficult by the poor prognosis of the disease, given its resistance to treatment and tumor recurrence after surgical excision. In addition, the inhibition of glioblastoma cell growth after treatment with hortensins 4 and 5 implies that RIPs could represent a possible adjuvant in combination with the normally used drug temozolomide.

On the other hand, the presence of type 1 RIPs in *A. hortensis* highlights that the Amaranthaceae family (previously known as Chenopodiaceae, order Caryophyllales) is a novel rich reservoir of these toxins, as confirmed by the isolation of similar enzymes in other species belonging to this family (e.g., *C. quinoa*, *S. soda*, *B. vulgaris* and *Amaranthus tricolor* L.). Because several publications have reported the possible involvement of these enzymes in plant stress responses, their knowledge could be useful for the agricultural system. Indeed, most Amaranthaceae species are intensively cultivated food crops, and research on these enzymes could help to improve yields, adaptability, and plant defense against pathogens and pests.

## 4. Materials and Methods

### 4.1. Materials

The material for chromatography was previously specified [19,20]. Single-stranded salmon sperm DNA was obtained from Sigma-Aldrich Solutions (Merk Life Science, Milan, Italy). The nuclease-treated rabbit reticulocyte lysate system was purchased from Promega (Madison, WI, USA). Other chemicals (analytical grade) were from Sigma-Aldrich Solutions.

Quinoin and sodin 5 from *C. quinoa and S. soda* seeds, respectively, used as reference type 1 RIPs, were isolated as previously reported [19,20].

The following buffers were used: buffer A: 5.0 mM Na-phosphate, pH 7.2, containing 0.14 M NaCl, buffer B: 10 mM Na-acetate, pH 4.0, and buffer C: 5.0 mM Na-phosphate pH 7.2.

### 4.2. Plant Seeds Samples

The seeds of *A. hortensis* var. *rubra* were purchased from Italian Sprout S.r.l., 47521-Cesena (https://www.italiansprout.com/, accessed on 4 September 2023). Seeds were separated from impurities and stored at −80 °C until further analyses.

### 4.3. Protein Purification

Type 1 RIP purification was conducted as previously reported [19,20,33,34]. Seeds (100 g) were homogenized in cold buffer A (1.0 L) using a Waring Blender (Waring Products, Torrington, CT, USA). The homogenate was stirred for 12 h at 4 °C and centrifuged at 15,000× *g* at 4 °C (1 h). The pH of crude extract was adjusted to pH 4.0 with glacial acetic acid, stirred at 4 °C for 1 h, and then centrifuged at 15,000× *g* at 4 °C (1 h). The supernatant was loaded onto a column (5.0 × 15 cm) containing Streamline^TM^ SP (Cytiva Italia S.r.l., Buccinasco (MI) Italy) equilibrated in buffer B at 3.0 mL/min flowrate. After sample loading, the resin was washed in buffer B and then in buffer C until the A_280_ nm was below 0.01 optical density (O.D.). Bound basic proteins were eluted with 1.0 M NaCl in buffer C, monitoring eluate absorbance at 280 nm. Fractions (10 mL) with absorbance at 280 nm were pooled and then concentrated in a cell concentrator (MWCO 10 kDa; Millipore Corporation, Danvers, MA, USA). These concentrated fractions, which contained basic proteins, were gel-filtered on a Hi-Load 26/60 Superdex^TM^ 75 (separation range 100–10 kDa; GE Healthcare, Milan, Italy), equilibrated and eluted with buffer C, containing 0.30 M NaCl, at a flow rate of 2.5 mL/min using the AKTA Purifier 100 FPLC (GE Healthcare). Fractions containing proteins with molecular weights of ~30 kDa were pooled, dialyzed against buffer C, and subjected to cationic exchange chromatographies. In particular, pooled fractions were subjected to cation exchange chromatography on CM-Sepharose Fast Flow (Cytiva; column L × I.D. 25 cm × 16 mm) equilibrated in buffer C and eluted with a 0.30 M NaCl linear gradient using a peristaltic pump (A: buffer C, 500 mL; B: buffer C containing 0.30 M NaCl, 500 mL; total volume 1 L). Subsequently, nonhomogeneous protein peaks after CM-Sepharose chromatography were pooled, dialyzed against buffer C, and further purified using cation exchange chromatography on a S-Sepharose Fast Flow column (Cytiva; column L × I.D. 20 cm × 12 mm), which was equilibrated with buffer C eluted with 0.30 M NaCl linear gradient using a peristaltic pump (A: buffer C, 300 mL; B: buffer C containing 0.30 M NaCl, 300 mL; total volume 0.6 L).

Purified enzymes were pooled, dialyzed against deionized water, freeze-dried, and stored at −20 °C until use.

### 4.4. Analytical Methods

Protein homogeneity was evaluated using SDS-PAGE with a Mini-Protean 3 (Bio-Rad, Milan, Italy) using a 6.0% stacking and 12% separating polyacrylamide gel with and without reducing agent [35]. The evaluation of 1D gel electrophoresis images was performed using GelAnalyzer software version 23.1 (http://www.gelanalyzer.com/?i=3, accessed on 7 February 2024). A Pierce BCA Protein Assay Kit (Life Technologies Italia Fil., Monza, Italy) was used to determine the protein concentration. Pro-Q™ Emerald 300 Glycoprot Probes Kombo (Life Technologies Italia) was used to determine the glycosylated proteins in gel analysis after SDS-PAGE. Glycosylated proteins were visualized using a ChemiDoc^TM^ XRS system (Bio-Rad).

### 4.5. Enzymatic Assays

The depurination assay (rRNA N-glycosylase assay) was conducted as previously described [20,22]. Briefly, aliquots (40 µL) of lysate from rabbit reticulocytes were incubated with 3.0 µg protein at 37 °C for 1 h. After treatment, RNA was extracted using phenolization, treated with 1.0 M acid aniline (pH 4.5), and precipitated. Finally, the RNA was subjected to electrophoresis at 15 mA for 2 h in a 7.0 M urea/5.0% (*w*/*v*) polyacrylamide gel and stained with ethidium bromide.

Polynucleotide:adenosine glycosidase activity on salmon sperm DNA (adenine release) was determined as previously reported [20,36].

RNase activity in polyacrylamide gel (zymogram) was performed as previously reported [37].

### 4.6. Cell-Free Protein Synthesis Inhibition

The inhibition of protein synthesis by *A. hortensis* protein fractions in a lysate of rabbit reticulocytes based on a bioluminescence assay was performed as previously described [36,38]. IC_50_ is the amount of protein that inhibits protein synthesis by 50% in a rabbit reticulocyte lysate system.

### 4.7. Cell Culture

U87MG human glioblastoma cells were purchased from Sigma-Aldrich Solutions (LGC Promochem, Teddington, UK). The cells were grown in Dulbecco’s modified Eagle medium (DMEM) in the presence of 10% fetal bovine serum (FBS), 2.0 mM L-glutamine, 100 IU mL^−1^ penicillin, and 100 µg mL^−1^ streptomycin and incubated at 37 °C, 5.0% CO_2_, and 95% humidity. In vitro experiments were performed with hortensin 4 and 5, which were purified as reported in paragraph 4.3, and their purity was evaluated using SDS-PAGE.

### 4.8. Dose–Response Effect of Hortensins 4 and 5 on U87MG Cells

To evaluate the dose–response effect of hortensins 4 and 5 on U87MG, cells were seeded into 96-well plates (5 × 10^3^ cells/well) and treated with increasing concentrations of hortensins 4 and 5 (0.001, 0.01, 0.1, and 1.0 µM) using only one induction. The effect of the toxins on the activities of mitochondrial dehydrogenase to convert the tetrazolium into formazan salts was estimated using an MTT assay. The MTT stock solution (5.0 mg mL^−1^) was dissolved in 1× phosphate buffer saline (PBS), and 10 µL were added to each well. Formazan crystals were solubilized using HCl containing 0.4% isopropanol, and the absorbance was measured at 570 nm. The dose–response data were plotted using GraphPad Prism 8 software (GraphPad Software Inc., San Diego, CA, USA).

### 4.9. Proliferation Assay

To evaluate the proliferation of U87MG cells as function of the hortensins 4 and 5 treatment, cells were seeded in 48-well plates (1 × 10^4^ cells/well) in DMEM in the presence of 10% FBS at 37 °C and 5.0% CO_2_. On the basis of the dose–response curves, cells were then treated daily with hortensins 4 and 5 at established concentrations of 0.1–1.0 µM for hortensins 4 and 0.1–0.5 µM for hortensins 5 for 24, 48, and 72 h. Cells were counted with a Burker chamber after 24, 48, and 72 h of treatment with both toxins.

### 4.10. Apoptosis Evaluation in U87MG Cell Line using DNA Fragmentation Detection through TUNEL Assay

The U87MG human glioblastoma cells were seeded in 8-well chamber slides (10 × 10^4^ cells/well) in DMEM supplemented with 0.5% FBS for 48 h and treated with hortensin 4, 0.1 and 0.5 µM, or hortensin 5, 0.1 and 1.0 µM, for 72 h. Subsequently, the cells were fixed for 25 min at 4 °C in a pH 7.4 solution of 4% methanol-free formaldehyde and washed two times with 1× PBS. The evaluation of DNA fragmentation was performed using a TUNEL assay using the commercial kit DeadEnd Fluorometric TUNEL System (Promega, Madison, WI, USA). Cells were counterstained with 4′,6-diamidino-2-phenylindole (DAPI) as mounting medium (Vectashield, LubioScience GmbH, Zürich, Switzerland) and analyzed using fluorescence microscopy, using Axiophot 2 Zeiss microscope (Zeiss, Göttingen, Germany) at 40× magnification.

### 4.11. Microscopic Observation of Live Cells

U87MG human glioblastoma cell lines were seeded in 96 wells in DMEM supplemented with 10% FBS and treated for 72 h with increasing concentrations (0.001, 0.01, 0.1, and 1.0 µM) of hortensins 4 and 5. Subsequently, cell morphological changes were evaluated using a phase contrast microscope (Evos, Life technologies, Monza, Italy) [32].

### 4.12. Statistical Analysis

Statistical analyses were performed using GraphPad Prism 8 software (GraphPad Software Inc., San Diego, CA, USA). For polynucleotide:adenosine glycosidase, the statistical significance was achieved using one-way ANOVA and a post hoc Dunnett’s test, while for the experiments on cells, statistical differences between groups were achieved using Student’s *t*-test or one-way ANOVA with a *p* < 0.05 considered statistically significant.

All the experiments performed in triplicate are expressed as mean ± SEM.

## Data Availability

The raw data supporting the conclusions of this article will be made available by the authors upon request, without undue reservation.

## Supplementary Materials

The following supporting information can be downloaded at https://www.mdpi.com/article/10.3390/toxins16030135/s1, Figure S1. SDS-PAGE analysis under reducing condition of peaks 1–5 fractions (3.0 µg each) after CM-Sepharose cation exchange chromatography (Figure 1a). M, molecular weight standards. SDS-PAGE was conducted on a 12% polyacrylamide separating gel in the presence of β-mercaptoethanol; Figure S2. Representative densitometric analysis of pooled 92–97 fractions from CM-Sepharose (peak 2; Figure 1a in main text) after SDS-PAGE under reducing conditions to determine the relative protein band amount. Densitometric analysis conducted on three different electropherograms, sampling aliquots of three pooled peaks 2 obtained using three different purifications; Figure S3. RNase zymography of hortensins 1, 2, 4, and 5 (lanes H1, H2, H4, and H5, respectively; 1.0 μg). Samples were separated using SDS-PAGE without reducing agent. The gel was stained using Toluidine Blue after incubation with total yeast RNA extract after washing to visualize RNA (blue background). RNase activity is indicated by the disappearance of RNA in the gel (white); Figure S4. Representative images from three independent experiments (n=3) after TUNEL assay of U87MG treated with hortensin 4 (H4; 0.1 and 0.5 μM) and hortensin 5 (H5; 0.1 and 1.0 μM) for 72 h compared with untreated U87MG (CT). Magnification 20×.
