# Supplementary material for: Hortensins, Type 1 Ribosome-Inactivating Proteins from Seeds of Red Mountain Spinach: Isolation, Characterization, and Their Effect on Glioblastoma Cells
Source: Toxins (Basel). 2024 Mar 4;16(3):135. doi: 10.3390/toxins16030135 (PMC10975204; doi:10.3390/toxins16030135)
Supplement: Supplementary file 1 [file toxins-16-00135-s001.zip › toxins-2807226-supplementary.pdf]

Supplementary Materials: Hortensins, Type 1 Ribosome-Inactivating Proteins from Seeds of Red Mountain Spinach: Isolation, Characterization, and Their Effect on Glioblastoma Cells

Sara Ragucci, Veronica Russo, Angela Clemente, Maria Giuseppina Campanile, Maria Antonietta Oliva, Nicola Landi, Paolo Vincenzo Pedone, Antonietta Arcella, Antimo Di Maro

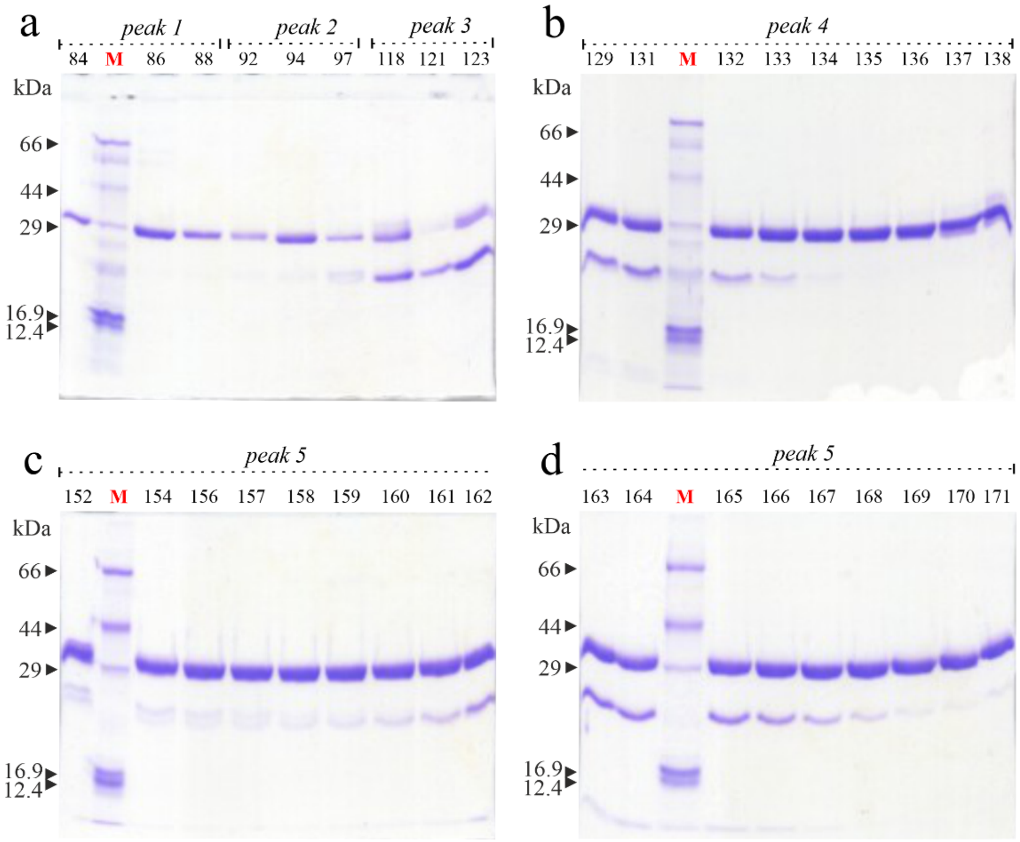

Figure S1. SDS-PAGE analysis under reducing condition of peaks 1-5 fractions (3.0 μg each) after CM-Sepharose cation exchange chromatography (Fig. 1a). M, molecular weight standards. SDS-PAGE was conducted on a 12% polyacrylamide separating gel in the presence of β-mercaptoethanol.

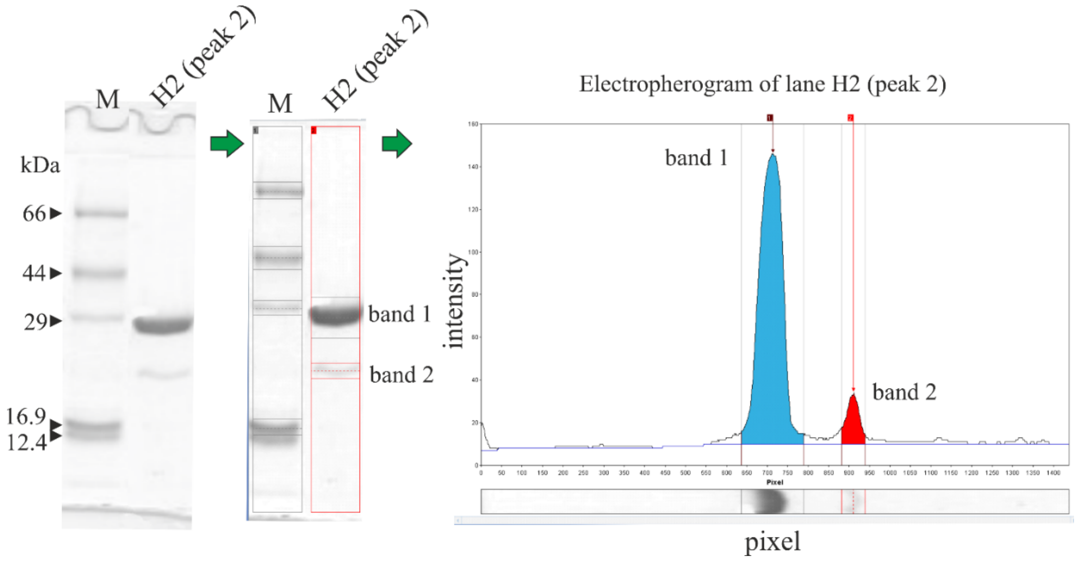

Figure S2. Representative densitometric analysis of pooled 92-97 fractions from CM-Sepharose (peak 2; Figure 1a in main text) after SDS-PAGE under reducing conditions to determine the relative protein band amount. Densitometric analysis conducted on three different electropherograms, sampling aliquots of three pooled peaks 2 obtained using three different purifications.

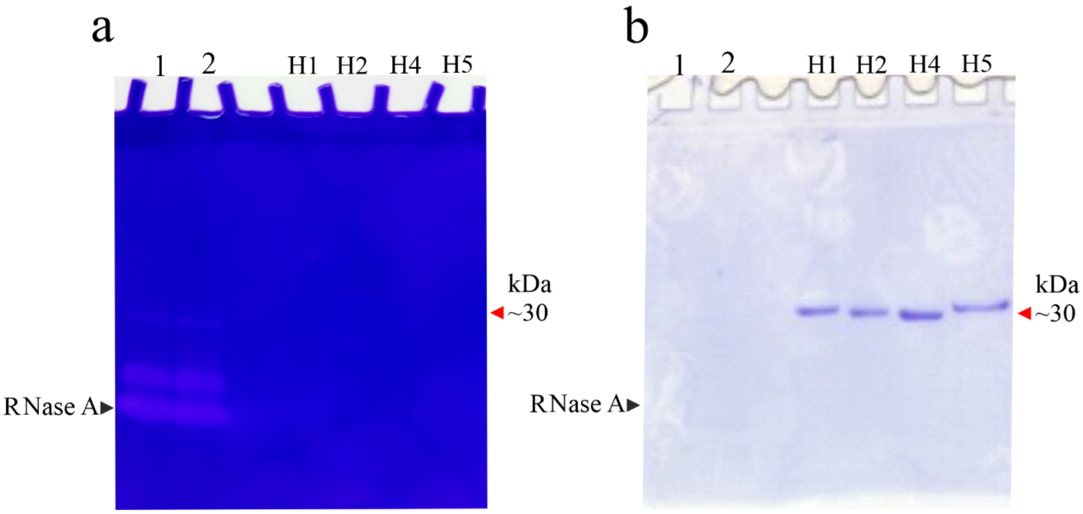

Figure S3. RNase zymogram and electrophoresis results of hortensins 1, 2, 4 and 5 (lanes H1, H2, H4 and H5, respectively; 1.0 μg). Samples were separated using SDS-PAGE without reducing agent. a) in gel staining using Toluidine blue after incubation with total yeast RNA extract and washing to visualize RNA (blue background). RNase activity is indicated by the disappearance of RNA in the gel (white). RNase A (lanes 1 and 2, 100 and 200 ng, respectively) was used as a positive control. b) Coomassie blue staining of gel in (a) after Toluidine blue removal.

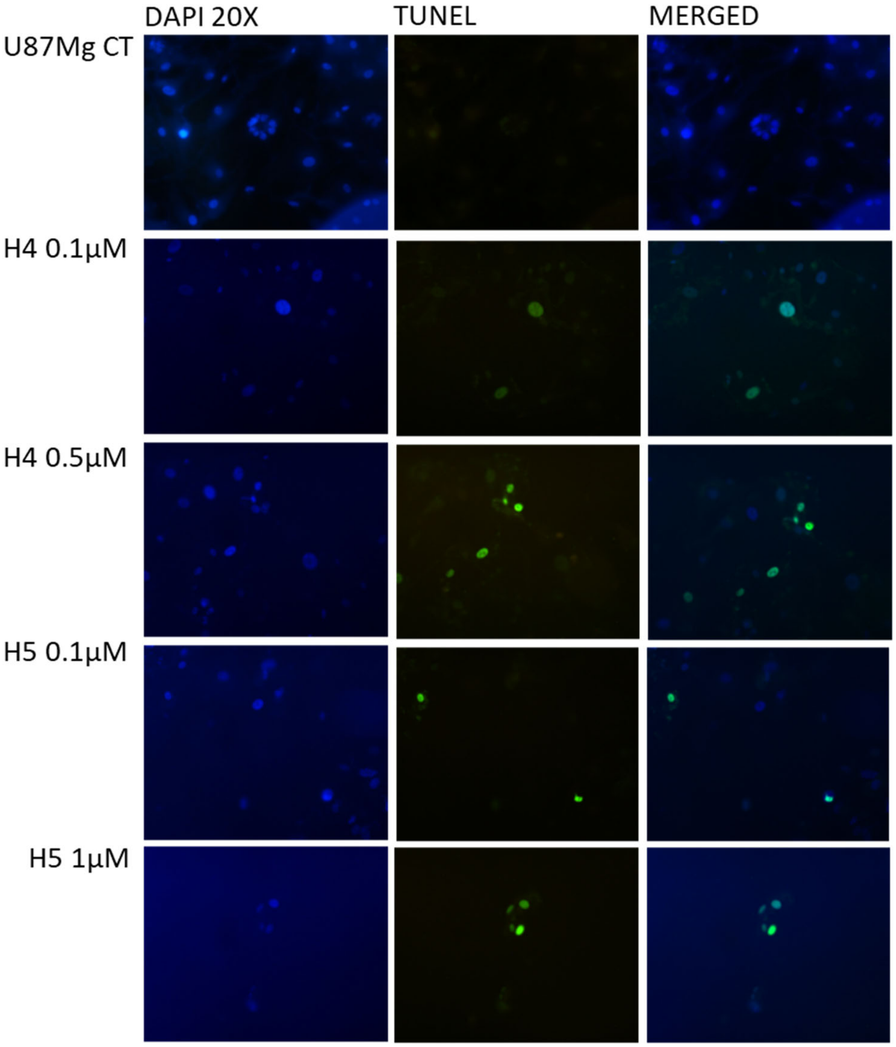

Figure S4. Representative images from three independent experiments (n=3) after TUNEL assay of U87MG treated with hortensin 4 (H4; 0.1 and 0.5 μM) and hortensin 5 (H5; 0.1 and 1.0 μM) for 72 h compared with untreated U87MG (CT). Magnification 20×.
